# Subtalar extra-articular screw arthroereisis (SESA) for the treatment of flexible flatfoot in children

**DOI:** 10.1007/s11832-014-0619-7

**Published:** 2014-11-21

**Authors:** Maurizio De Pellegrin, Désirée Moharamzadeh, Walter Michael Strobl, Rainer Biedermann, Christian Tschauner, Thomas Wirth

**Affiliations:** 1 Pediatric Orthopedic Unit, San Raffaele Hospital, Via Olgettina 60, Milan, Italy; 2 Department Pediatric Orthopedics, Orthopedic Hospital Speising, Speisingerstrasse 109, 1130 Vienna, Austria; 3 Department of Orthopedics, Medical University of Innsbrück, Anichstr. 35, 6020 Innsbruck, Austria; 4 Landeskrankenhaus (LKH) Stolzalpe, Stolzalpe 38, 8852 Stolzalpe, Austria; 5 Pediatric Orthopedics Center, Olgahospital, Bismarckstr. 8, Stuttgart, Germany

**Keywords:** Flexible flatfoot, Arthroereisis, Calcaneo-stop, Minimally invasive surgery

## Abstract

**Purpose:**

The aim of this study was to describe a subtalar extra-articular screw arthroereisis (SESA) technique for the correction of flexible flatfoot (FFF) in children and report the outcome.

**Methods:**

From 1990 to 2012, data were collected on 485 patients who underwent SESA at the San Raffaele Hospital. The average age of the patient cohort was 11.5 ± 1.81 years (range 5.0–17.9 years; median 11.5 years). Inclusion criteria were FFF and marked flexible hindfoot valgus, and the exclusion criterion was rigid flatfoot. SESA was performed in 732 cases of FFF—bilaterally in 247 patients and monolaterally in 238 patients.

**Results:**

The values of the pre- and post-SESA weight-bearing X-ray angles were 146° ± 7° and 129° ± 5°, respectively, for the Costa-Bartani angle, 43° ± 8° and 25° ± 6°, respectively, for the talar inclination angle and 11° ± 6° and 14° ± 5°, respectively, for calcaneal pitch (*p* <0.001). All data were analysed statistically with Student’s *t* test. Data on 398 patients were ultimately available for analysis. In 93.7 % of cases the results were good in terms of improved clinical aspects and X-ray measurement, absence of complications, normal foot function 3 months post-SESA and no requirement for further surgery. The complication rate was 6.3 % and included ankle joint effusion, painful contracture of peroneal muscles and fourth metatarsal bone stress fractures. A sample of 76 patients (121 feet) were evaluated after screw removal, which occurred on average 2.9 years after SESA. The angle measurements of this sample showed no statistically significant modification.

**Conclusion:**

Based on our >20 years of experience, we believe that SESA is an optimal technique for the correction of FFF as it is simple and can be performed rapidly, and the corrective effect results from the screw’s mechanical and proprioceptive effect. The indication for surgery must be accurate. We suggest that the patient be at least 10 years of age in order that all of the foot’s growth potential can be utilized and to allow for spontaneous resolution and thereby avoid the possibility of over-treatment.

## Introduction

Although flexible flatfoot (FFF) is undoubtedly one of the most frequent skeletal disorders in children, little is known about its incidence. Definitions vary, and there is as yet no general consensus on the level of change which marks the end of variations in normal foot shape and the start of foot deformity [1, 2]. An abnormally low or absent arch, an excessive eversion of the heel during weight-bearing and an abducted forefoot producing a midfoot sag are the usual criteria of this condition. In children with FFF, the longitudinal arch will reconstitute when the child is standing on tiptoe or there will be hyperextension of the great toe due to the windlass mechanism of the plantar fascia.

Asymptomatic FFF is almost a universal finding in toddlers due to presence of subcutaneous fat which will resolve spontaneously [3, 4], and its prevalence in children progressively decreases with age [5]. Harris [3] reported that the prevalence of FFF is 54 % at 3 years of age, falling to 24 % between 3 and 6 years of age. In a study of pre-school aged children, Sullivan [6] reported a prevalence of FFF in 52 % of the boys and 36 % of the girls. Staheli et al. [7, 8] demonstrated that most infants exhibit flat feet and that the longitudinal arch develops within the first decade of life. These observations were confirmed by Volpon [9] who reported that, in most cases, the footprint at 6 years of age corresponds to that of the adult.

FFF rarely causes pain or disability in infancy and childhood [1]. Children usually present to the out-patient clinic because of parents’ concern on their foot appearance and/or excessive asymmetric shoe wear [5]. There is broad consensus that the “typical” case of an asymptomatic paediatric patient with FFF needs no specific treatment except for “wait and watch” [1–3, 7, 10]. If the clinical examination reveals an apparent shortening of the Achilles, then stretching exercises are indicated. However, it has been reported that a FFF which develops a retraction of the Achilles tendon will certainly worsen in adult life and will become symptomatic [3, 11–13]. To date there are no long-term prospective studies on the natural history of untreated FFF [1], but it has been suggested that in its late stage of progression degenerative arthritis may occur, leading to the loss of flexibility and ankylosis [14, 15]. An increasingly frequent pattern of tibialis posterior overuse has also been described in some studies, mainly correlated to a pre-existent foot deformity in childhood [16, 17]. For symptomatic patients, inlays or even orthoses are sometimes recommended. However, the study of Wenger and Leach [18] and the recent literature review of MacKenzie et al. [17] on the effect of paediatric foot orthoses found very limited evidence on the effectiveness of non-surgical interventions in children with FFF. Therefore, in symptomatic patients who are unresponsive to conservative measures, surgery is often considered.

Interestingly, although there is a reluctance in the American literature to classify ‘flexible flatfoot’ as a distinct nosological entity in need of a surgical treatment, there have been ample descriptions in reference books of invasive techniques for its treatment [19]. Here, we describe a mini-invasive technique which has been adopted and routinely performed at the medical institutions of the authors since 1989 [20] for the treatment of FFF in children. The procedure has a low complication rate, a low surgical risk and is reversible in case of failure. The aim of our study is to describe this technique, referred to as the subtalar extra-articular screw arthroereisis (SESA) technique, and to analyse the outcomes.

## Materials and methods

Between 1990 and 2012, data on the 485 patients (267 males; 218 females) who underwent SESA at the Paediatric Orthopaedic Unit of San Raffaele Hospital (Milan, Italy) were collected. The inclusion criteria of this study were 1 FFF with a valgus hindfoot (*pes plano valgus*) and marked flexible valgus of the hindfoot (*pes calcaneus valgus*).

At our five medical institutions, the clinical assessment of a child with flatfoot consists of a medical examination of the foot, a footprint analysis and X-ray measurements. The aim of the general medical examination is to assess torsional and angular variations of the lower extremities, walking pattern and evidence of generalised ligamentous laxity. FFF may cause rapid and uneven shoe wear in older children and adolescents, and so the child’s shoes are also examined. A flatfoot is a combination of deformities [1] of which the main aspect is the valgus position of the calcaneus, which leads to a medial, plantar tilt of the talus [21] and, therefore, a reduction or absence of the longitudinal arch. Ankle dorsiflexion and Achilles tendon excursion are evaluated as there may also be a contracture of the gastrocnemius or the entire triceps’ surae. Nevertheless, the most important target of the clinical assessment is the flexibility of the flatfoot, rather than its static shape [1]. All footprints were defined according to the Staheli Arch Index [22]. Pre- and post-SESA weight-bearing X-ray angles were measured, including the Costa-Bartani angle, talar inclination angle and calcaneal pitch angle [23].

Indications for surgery were painful FFF; FFF with Achilles tendon shortening (positive Silverskiöld test); Staheli Arch Index >1; pathological values for two of three weight-bearing X-ray angles defined above.

### SESA: surgical technique

**Fig. 1:**
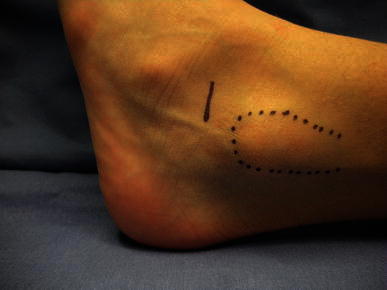
The minimally invasive skin incision at the level of the sinus tarsi is approximately 1.5 cm

Since 1989 we have adopted the original technique proposed by Recaredo Álvarez in 1972, with the only modification being the substitution of the cortical screw with a cancellous screw (as previously suggested by Pisani [24]). The technique requires a mini-invasive incision (Fig. 1), followed by manual reduction of the talo-calcaneal derotation which is then kept in the correct position by means of a screw inserted at the level of the sinus tarsi, under the talus lateral process. The correction is verified by observing the position of the hindfoot with respect to the longitudinal axis of the leg: if a foot is properly corrected, both malleoli will be seen from a plantar view (“Plantar Malleoli View Sign”). If undercorrected, the medial malleolus is visible, and the screw has to be pulled out a few threads from the calcaneus so that it protrudes slightly more; vice versa, if overcorrected only the lateral malleolus is seen, and the screw has to be inserted further into the calcaneus. Partial weight-bearing is allowed 5 days post-surgery, with complete weight-bearing by postoperative day 11. Sport activities are forbidden for 1 month. No cast is required.

The technique is contraindicated when it is impossible to perform a manual reduction due to, for example, rigid flatfoot, post-traumatic flatfoot, talo-calcaneal coalitio, congenital vertical talus, among others.

A possible alternative to the technique is to substitute the AO cancellous screw with a dedicated, self-threading, cannulated screw.

Among our patients, SESA was performed in 732 cases of FFF—bilaterally in 247 patients (143 males; 104 females) and monolaterally in 238 patients [118 cases (69 males; 49 females) of right foot involvement and 120 cases (55 males; 65 female) of left foot involvement]. In the bilateral cases surgery was performed on one foot first; the second foot was operated on an average of 6 months after the first, if the indication was still present. The average (± standard deviation) age at first surgery was 11.5 ± 1.81 (range 5.0–17.9; median age 11.5) years. The average follow-up was 4.5 (range 3.1–13.2) years.

To analyse the outcomes, we collected data on 138 patients (227 feet) who underwent removal of the screw an average of 3.1 years after SESA and then evaluated the X-ray and clinical outcome after removal in a subsample of 76 of these patients (121 feet). The time of surgery of this sample was on average 2.9 years after SESA, and the average age at surgery was 13.51 ± 1.80 (median age 14) years.

All data were subjected to statistical analysis with the Student *t* test.

## Results

All patients were clinically evaluated at pre-determined post-operative time-points: 5, 11 and 30 days; 3 and 6 months; 1 and 2 years; at the time of screw removal; 1 year following screw removal. All patients achieved full weight-bearing between postoperative days 8 and 11. With the exception of cases with complications (discussed below), all patients achieved a correction of the hindfoot valgus in the immediate postoperative period, although the clinical heel valgus angle was not measured during this period to avoid a subjective interpretation. The pre- and post-SESA clinical parameters evaluated are described in Table 1.

**Table 1:** Pre- and post-SESA (3 months postoperative) clinical evaluation data for 485 patients

Parameter assessed	Time-point of clinical evaluation	
	Pre-SESA (%)	Post-SESA (%)
Fatigue	16	3
Pain	11	2
Tripping	8	49
Orthoses	67	11
Forefoot abductus	65	0
Forefoot adductus	0	15
Valgus hindfoot	100	0
Varus hindfoot	0	7

*SESA*Subtalar extra-articular screw arthroereisis

There were no cases of superficial or deep wound infection or of decubitus of the implanted screw. No patient underwent antibiotic prophylaxis unless required for another pre-existent or potential systemic disease (diabetes, endocarditis).

The pre- and post-SESA weight-bearing X-ray angle measurements (Costa-Bartani angle, talar inclination angle, calcaneal pitch angle) are shown in Table 2. The results show a statistically significant (*p* <0.001) improvement in the post-operative measurements of all angles (Costa-Bartani 129° ± 5°; talar inclination 25° ± 6°; calcaneal pitch 14° ± 5°). Table 2 also reports the pre- and post-operative weight-bearing X-ray angle measurements after screw removal. These results demonstrate that after screw removal, there was no statistically significant modification of the post-operative angle measurements (average: Costa-Bartani 122.9°; talar inclination 19.8°; calcaneal pitch 19.6°).

**Table 2:** X-ray angle measurements pre- and post-SESA and at screw removal

Weight-bearing X-ray angles	SESA		Screw removal	
	Pre-operative (first surgery)	Post-operative (first surgery)	Pre-operative (average)	Post-operative (average)
Costa-Bartani angle (N.V. 120°–125°)	146° ± 7°	129° ± 5°	123.9°	122.9°
Talar inclination angle (N.V. 15°–20°)	43° ± 8°	25° ± 6°	19.3°	19.8°
Calcaneal pitch angle (N.V. 20°–30°)	11° ± 6°	14° ± 5°	20.2°	19.6°

*N.V.* Normal variation

Nine patients underwent a second surgery (substitution or regularisation of the screw) for a loss of correction. In seven patients the loss of correction was due to a growth spurt, as the indication had been given at an earlier age (age range at first surgery 5–9 years) due to the severity of the FFF. In the two patients, the indication to substitution (*n* = 1) and to regularisation (*n* = 1) were given for an abnormal growth of the foot with a loss of the mechanical effect of the screw.

During the period 2004 and 2012 data was collected on complications in 25 (6.3 %) of 398 patients. The patients with complications were categorized as follows:

- Group A (*n* = 8): ankle joint effusion or haemarthrosis, in absence of acute inflammatory signs, with an important decrease in the range of motion and painful weight-bearing;
- Group B (*n* = 14): contracture of the peroneal muscles due to an antalgic position in pronation;
- Group C (*n* = 3): stress fractures of the fourth metatarsal bones due to abnormal gait with excessive weight-bearing on the fourth to fifth rays.

The treatment of patients categorized in Group A was early removal of the screw (average 8 months post-SESA) when conservative treatment had been unsuccessful. At revision surgery, it became apparent that during the intra-operative period all patients had an osteolysis of the talus lateral process (where there was contact with the screw’s head). A new screw was successfully implanted in two patients 1 year later, one patient was treated elsewhere and five patients did not undergo further surgery and the correction therefore obtained remained partial.

For three Group B patients, the treatment consisted of a cast in full supination for 2 weeks, followed by physiotherapy, with plantar orthoses prescribed until symptom resolution. In six Group B patients symptom resolution was obtained with physiotherapy and plantar orthoses only. Five Group B patients required two injections of methylprednisolone acetate (1 ml) locally at the level of the screw head to obtain symptom resolution.

**Fig. 2:**
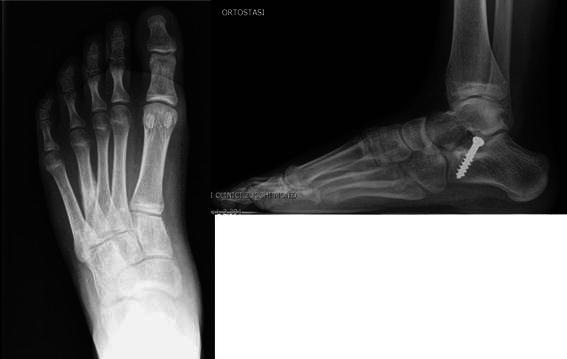
Stress fracture of the fourth metatarsal bone in a patient who underwent subtalar extra-articular screw arthroereisis (SESA). The patient mentioned pain along the lateral border of the midfoot which an X-ray 3 months after surgery confirmed to be due to a healed stress fracture

The three patients in Group C received no treatment as when they presented at the out-patient clinic (an average of 3 months after surgery) mentioning pain along the lateral border of the midfoot, an X-ray revealed a healed fracture of the fourth metatarsal bone in all cases (Fig. 2).

**Fig. 3:**
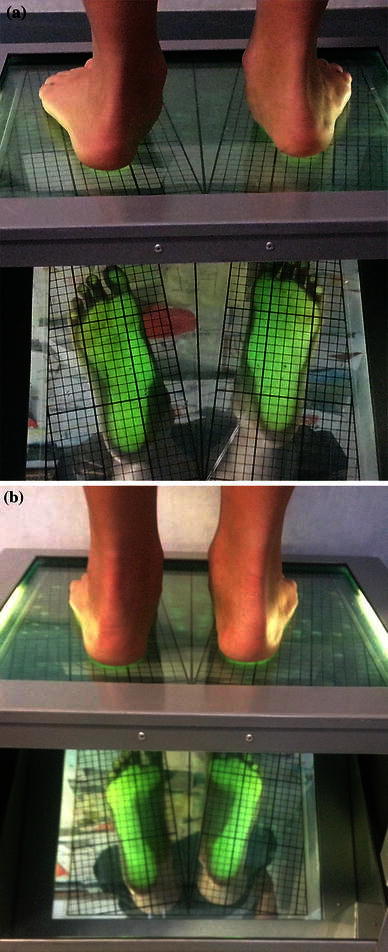
Clinical aspect and footprint analysis of a 12-year old boy with a bilateral flexible flatfoot. **a** pre-SESA, **b** post-SESA

**Fig. 4:**
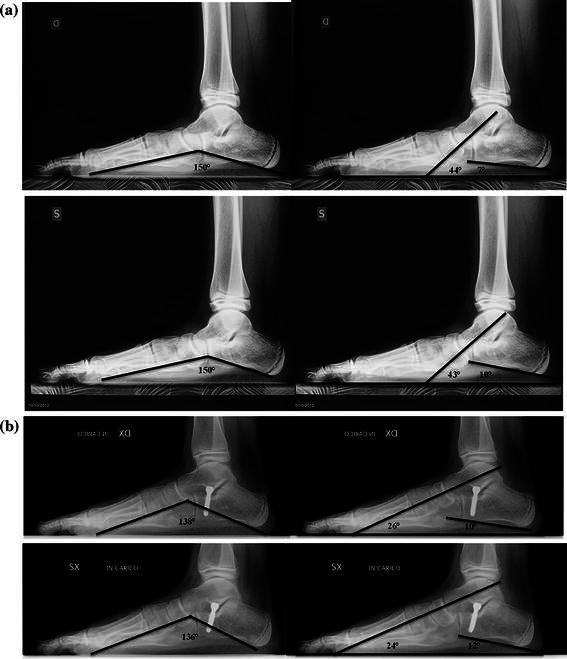
X-ray at weight-bearing of the same patient in Fig. 3 with measurements of Costa-Bartani angle (*left*) and talar inclination and calcaneal pitch angles (*right*). **a** pre-SESA, **b** post-SESA

Our results were good in 93.7 % of cases in terms of clinical outcome (Fig. 3a, b), radiographic improvement (Fig. 4a, b), complication rate and foot function 3-months post-SESA. The data are summarised in Table 3.

**Table 3:** Summary of clinical and radiographic outcomes

Clinical and radiographic outcomes	
Good outcome (93.7 %)	Poor outcome (6.3 %)
Improvement of clinical aspects and x-ray angles	No improvement
No complications	Complications
Normal foot function 3 months post-SESA	Pathological foot function 3 months post-SESA
No further surgery	Additional surgery required

## Discussion

In the intervening years since Recaredo Álvarez proposed the original subtalar arthroereisis technique in 1972, many different variations have been reported. In this study we refer only to variations in the extra-articular (SESA) technique, leaving the discussion on techniques inside the sinus tarsi and comparisons to other authors.

The surgical indications for FFF have to be rigorous, especially as the distinction between a physiological and a pathological situation is not always clear-cut. Several authors [11–13] have reported that the physiological valgus of the child’s foot spontaneously evolves to the shape of the adult’s foot at around 10 years of age and that thereafter there are no important changes of the foot’s longitudinal arch and its global shape. Based on these studies, we therefore recommend waiting to see whether the foot develops normally—until the child is at least 10 years old—before proceeding with a surgical correction.

Numerous surgical procedures for the correction of FFF have been proposed. These can be categorised as soft tissue plications, tendon lengthening and transfers, osseous excisions, osteotomies, arthrodesis of one or more joints and the interposition of bone or synthetic implants into the sinus tarsi [14, 25–34].

In 1946, Chambers [35, 36] described for the first time the concept of arthroereisis for pathologic pronation of the foot. He believed that the excessive eversion would be limited by elevating the sinus tarsi floor with an autogenous bone graft under the leading edge of the calcaneus posterior facet. In 1970, LeLièvre [35] employed autogenous bone grafts within the sinus tarsi to limit pronation, using a free-floating bone graft obtained from the base of the proximal phalanx, which was then resected as part of the hallux valgus repair. This author was the first to introduce the term “lateral arthroereisis” [35]. In 1983, Smith and Millar [37] first described the subtalar arthroereisis-peg procedure, using a device made of ultrahigh molecular-weight polyethylene. Other devices which have been used include a threaded polyethylene plug inserted into the sinus tarsi, described by Giannini in 1985 [38], and a champagne glass-shaped silicon implant, described by Viladot in 1992 [21].

In the 1980s, Pisani et al. [24, 39] introduced into Italy the technique suggested by Recaredo Álvarez in Spain in 1970, subsequently published by Burutaran in 1979 [40]. This technique, also known as “calcaneo-stop”, is an extra-articular arthroereisis of the subtalar joint and therefore performed outside the sinus tarsi. Its general application quickly spread throughout Italy [20, 41–45] and, more recently, into other European countries [20, 46–48]. Although many different variations of the original technique have appeared since its introduction, the principles of the correction are still the same [41, 49].

Magnan et al. [42] compared the original technique and Castaman’s modified technique [41] and found that the mechanical component of the technique was more important than the type of screw implanted. In an Italian study [43], 306 patients affected by flatfeet (480 feet) underwent surgery with the either Alvarez technique or Castaman technique (in which the screw is implanted in the talus instead of the calcaneus). The screws used were AO screws (cortical or cancellous). In the Alvarez arm of this study, both types of screw were used, while in the Castaman arm, only the cancellous screws were implanted. In those patients treated with the Alvarez technique, the authors observed no significant differences in the results and complications (loosening, osteolysis, rupture of the screws) between the cancellous (diameter 4.5/6.5 mm) and cortical (diameter 3.0/4.5 mm) screws. In contrast, in those patients treated with the Castaman technique, rupture of the cancellous screw implanted in the talus was reported in 6.3 % of cases. These results imply that the position and mechanical action of the implant is fundamental to the success of the technique—and not the type of implant itself. However, the action of the screw in the original technique is presumed to be more than just mechanical, as suggested in our study where there were only a few cases of loosening of the screw and osteolysis of the talus lateral process (in long term follow-up) was absent in the great majority of patients. Moreover, in a few cases of bilateral involvement, we observed a spontaneous correction of the non-operated foot prior to surgery on the contra-lateral. The mechanism underlying this correction is assumed to be the proprioceptive one.

How does the screw work? First, we know it has a mechanical effect because the result is immediate: in the younger children enrolled in our study the correction decreased with growth and in eight cases reported here there was a protrusion of the screw head into the talus, where contact is the greatest. Second, it is known that joint stability is constituted by static and dynamic elements, with the former depending on the anatomical congruity of joint surfaces and on ligamentous restraints which limit joint translations. In contrast, the dynamic joint stability implies a proprioceptive control of the compressive and directional muscular forces which act on the joint [50]. Proprioception plays a critical role in ankle joint stability; in particular, the subtalar joint has a critical function in adapting the foot to the ground [51].

The role of proprioception post-SESA has been suggested in previous studies [24, 41]. Based on the analysis of our 23-year data set, we agree with this theory as we encountered a number of interesting aspects in our patients. During our clinical experience, we have also performed SESA in patients with osteogenesis imperfecta [20, 52], who have a lower bone resistance, and found no signs of screw protrusion in these patients. Another aspect we considered is that the screw becomes shorter during foot growth, as seen at removal surgery; however, as the correction is persistent in most cases, another type of correction rather than the mechanical one is implied. Furthermore, in 14 patients we noted a peroneal contracture, which is a reaction to pain and to stimulation of the sinus tarsi mechanoreceptors, as described in the following text.

Rein et al. [53] analysed the pattern and types of mechanoreceptors (Ruffini endings, Pacini corpuscles, Golgi-like endings, free nerve endings and unclassifiable corpuscles) in the different anatomical complexes of ankle ligaments using designated immunohistochemical markers. The free nerve endings were the predominant mechanoreptor type, followed by Ruffini endings, indicating that nociception and joint position are greatly important in terms of ankle proprioception. In a following study, Rein et al. [54] showed that the lateral root of the inferior extensor retinaculum at the entrance of the sinus tarsi was richly innervated with free nerve endings, as compared to the deeper situated canalis tarsi ligament. Based these observations, it may be assumed that the pain of the sinus tarsi syndrome mainly originates at the entrance of this structure. Other studies have shown that patients with functional ankle instability and pain near the sinus tarsi have a prolonged peroneal reaction time (PRT) [55]. This prolonged PRT suggests a proprioceptive role of the sensory nerve endings at the sinus tarsi in regulating the activities of the gamma motor neurons of the peroneal muscles, which in turn may cause the symptoms of functional ankle instability and prolonged PRT. These studies by Rein et al. [53, 54] provide the basis to explain how the screw works at the level of the lateral subtalar joint, below the talar lateral process, by explaining the proprioceptive effect of the screw on one hand and possibly elucidating those cases of peroneal muscle contracture which have no identifiable failure of the surgical technique on the other hand. An interesting research question in the framework of comparing inside and outside sinus tarsi devices would be to examine how a device implanted inside the canalis tarsi can stimulate the receptors (both in a mechanical and proprioceptive manner).

Among the patients with FFF treated with SESA in our study, the clinical and X-ray studies during the follow-up period show good outcomes in approximately 94 % of patients, even after screw removal. Our X-ray measurements show a greater improvement of the Costa-Bartani and talar inclination angles than of the calcaneal pitch angle, probably due to the site of correction, i.e. the subtalar joint. The average follow-up was 4.5 years, and, at the time of evaluation all our patients had reached complete foot skeletal maturity and had no recurrence of the deformity. We evaluated the outcome in 121 feet after screw removal, which occurred on average 2.9 years post-SESA. The data collected after screw removal show similar pre- and post-operative values of the Costa-Bartani and talar inclination angles, which, however, are improved with respect to the measurements post-SESA. Interestingly, the calcaneal pitch angle at screw removal, in contrast to the immediate post-SESA period, did improve, although in a statistically nonsignificant manner. This result may be explained by the calcaneus progressive correction after the improvement of the talo-calcaneal relationship at the subtalar joint and demonstrates that the correction obtained with SESA is effective, progressive and maintained.

In our population there was never the need to perform a gastrocnemuis recession in idiopathic FFF, although Achilles tendon retraction in FFF was an indication for surgery; physiotherapy has proved to be an effective treatment after SESA. The term “flexible flatfoot” implies a deformity which can actively be corrected in all planes when the patient is on tiptoes and manually during the examination. The concomitant presence of an Achilles tendon retraction before surgery does not limit the “flexibility” of the deformity, but it may determine the absence of a future spontaneous correction. However, in our patients, if the retraction was present it did not modify our surgical procedure.

Approximately 15 % of our patients had an in-toeing gait and a foot in the supination position for the first 3 months, which we did not consider as complications. The actual complication rate among our patients was 6.3 % and includes patients with ankle joint effusion or haemarthrosis, contracture of the peroneal muscles due to an antalgic position in pronation and stress fractures of the fourth metatarsal bone due to an abnormal gait with excessive weight-bearing on the fourth to fifth rays. Clearly fracture of the fourth metatarsal occurred, as the fifth ray is physiologically more mobile. These patients were all treated accordingly and symptom resolution occurred in most cases.

One limitation of this study was the lack of a patient’s satisfaction survey and a validated evaluation score after surgery. However, during the long follow-up period, nearly all patients were clinically and functionally satisfied with the outcomes. The indication for surgery was given after the patient had reached an age of 10 years, following which, as reported in the literature, there will be no further spontaneous improvement in the natural history of FFF occurs. Therefore, another limitation to our study was the lack of a control group of children with FFF with surgical indication who were aged >10 years and had not undergone surgery.

In approximately 50 % of our patients there was a monolateral involvement. Interestingly, in the majority of these patients the clinical findings were markedly “monolateral”. The definition of “pathologic” is much clearer in these cases with respect to patients with bilateral involvement, where the concept of “physiological variant” may arise.

## References

[1] Mosca VS: (2010). Flexible flatfoot in children and adolescents. J Child Orthop.

[2] Herring JA (2002) Flexible flatfoot (pes calcaneovalgus). In: Herring JA, Tachdjian MO; Texas Scottish Rite Hospital for Children. Tachdijan’s pediatric orthopedics. Saunders/Elsevier Health Sciences, Amsterdam, pp 908–921

[3] Harris EJ (2010). The natural history and pathophysiology of flexible flatfoot. Clin Pod Med Surg.

[4] Sullivan JA (1999). Pediatric flatfoot: evaluation and management. J Am Acad Orthop Surg.

[5] Rao UB, Joseph B (1992). The influence of footwear on the prevalence of flat foot. A survey of 2,300 children. J Bone Jt Surg Br.

[6] Pfeiffer M, Kotz R, Ledl T (2006). Prevalence of flat foot in preschool-aged children. Pediatrics.

[7] Staheli LT, Chew DE, Corbett M (1987). The longitudinal arch. A survey of eight hundred and eighty-two feet in normal children and adults. J Bone Jt Surg Am.

[8] Forriol F, Pascual J (1990). Footprint analysis between three and seventeen years of age. Foot Ankle.

[9] Volpon JB (1994). Footprint analysis during the growth period. J Pediatr Orthop.

[10] McCarthy DJ (1989). The developmental anatomy of pes valgo planus. Clin Podiatr Med Surg.

[11] Hefti F (1997). Kinderorthopädie in der Praxis.

[12] Jani L (1986). Der Kindliche Knick-Senkfuss. Orthopäde.

[13] Staheli LT (2003). Fundamentals of pediatric orthopedics.

[14] Lee MS, Vanore JV, Thomas JL (2005). Clinical practice guideline adult flatfoot panel. Diagnosis and treatment of adult flatfoot. J Foot Ankle Surg.

[15] Pinney SJ, Lin SS (2006). Current concept review: acquired adult flatfoot deformity. Foot Ankle Int.

[16] Bohay DR, Anderson JG (2003). Stage IV posterior tibial tendon insufficiency: the tilted ankle. Foot Ankle Clin.

[17] MacKenzie AJ, Rome K, Evans AM (2012). The efficacy of nonsurgical interventions for paediatric flexible flat foot: a critical review. J Pediatr Orthop.

[18] Wenger DR, Leach J (1986). Foot deformities in infants and children. Pediatr Clin North Am.

[19] Jay RM (1999). Pediatric foot and ankle surgery.

[20] De Pellegrin M (2005). Die subtalare Schrauben-Arthrorise beim kindlichen Plattfuß. Orthopäde.

[21] Viladot A (1992). Surgical treatment of the child’s flatfoot. Clin Orthop Relat Res.

[22] Staheli T, Deanna E Chew, Corbett M (1987) The longitudinal arch. J Bone Jt Surg 69:426–428 3818704

[23] Gasparini G, Espa E, Pianezzi M, De Santis E (1993) Il piede piatto. Semeiotica strumentale: RX e TC. Aulo Gaggi, Bologna

[24] Pisani G (1993) Piede calcaneo-valgo. In: Trattato di chirurgia del piede. Minerva Medica, Torino, pp 243–250

[25] Addante JB, Chin MW, Loomis JC (1992). Subtalar joint arthroereisis with SILASTIC silicone sphere: a retrospective study. J Foot Surg.

[26] Adelman VR, Szczepanski JA, Adelman RP (2008). Radiographic evaluation of endoscopic gastrocnemius recession, subtalar joint arthroereisis, and flexor tendon transfer for surgical correction of stage II posterior tibial tendon dysfunction: a pilot study. J Foot Ankle Surg.

[27] Brancheau SP, Walker KM, Northcutt DR (2012). An analysis of outcomes after use of the Maxwell-Brancheau Arthroereisis implant. J Foot Ankle Surg.

[28] Dragonetti L, Ingraffia C, Stellari F (1997). The young tenosuspension in the treatment of abnormal pronation of the foot. J Foot Ankle Surg.

[29] Forg P, Feldman K, Flake E, Green DR (2001). Flake-Austin modification of the STA-Peg arthroereisis: a retrospective study. J Am Podiatr Med Assoc.

[30] Giorgini RJ, Schiraldi FG, Hernandez PA (1988). Subtalar arthroereisis: a combined technique. J Foot Surg.

[31] Jacobs AM (2007). Soft tissue procedures for the stabilization of medial arch pathology in the management of flexible flatfoot deformity. Clin Podiatr Med Surg.

[32] Koning PM, Heesterbeek PJ, de Visser E (2009). Subtalar arthroereisis for pediatric pes planovalgus: fifteen years experience with the cone-shaped implant. J Am Podiatr Med Assoc.

[33] Kwon JY, Myerson MS (2010). Management of the flexible flat foot in the child: a focus on the use of osteotomies for correction. Foot Ankle Clin.

[34] Metcalfe SA, Bowling FL, Reeves ND (2011). Subtalar joint arthroereisis in the management of pediatric flexible flatfoot: a critical review of the literature. Foot Ankle Int.

[35] LeLièvre J (1970). Current concepts and correction of the valgus foot. Clin Orthop Relat Res.

[36] Peters PA, Sammarco J (1989). Arthroereisis of the subtalar joint. Foot Ankle Clin.

[37] Smith SD, Millar EA (1983). Arthroereisis by means of the subtalar polyethylene peg implant for correction of hindfoot pronation in children. Clin Orthop Relat Res.

[38] Giannini S, Girolami M, Ceccarelli F (1985). The surgical treatment of infantile flatfoot. A new expanding endo-orthotic implant. Ital J Traumatol.

[39] Milano L, Scala A (1985). La risi extrarticolare della sottoastragalica con endortesi calcaneale nel trattamento chirurgico delle deformità in valgo del calcagno. Chir Piede.

[40] Burutaran JM (1979). El calcaneo-stop para el tratiamento del valgo de talon infantile. Chir Piede.

[41] Castaman E (1985). L’intervento di calcaneo-stop nel piede piatto valgo. Chir Piede.

[42] Magnan B (1997). Flatfeet: comparison of surgical techniques. Results of study group into retrograde endorthesis with calcaneus-stop. Ital J Ped Orthop.

[43] De Pellegrin et al (1997) Il piede piatto: tecniche chirurgiche a confronto. Risultati del gruppo di studio sul calcaneo-stop con impiego delle viti AO. Riv Ital Ortop Traumatol Ped 13:231–237

[44] Usuelli FG, Montrasio UA (2012). The calcaneo-stop procedure. Foot Ankle Clin.

[45] Pavone V, Costarella L, Testa G (2013). Calcaneo-stop procedure in the treatment of the juvenile symptomatic flatfoot. J Foot Ankle Surg.

[46] De Pellegrin M (2007). Subtalar screw arthroereisis for correction of flat foot in children—15 years experience. Fuss und Sprungelenk.

[47] Roth S, Sestan B, Tudor A (2007). Minimally invasive calcaneo-stop method for idiopathic flexible pes planovalgus in children. Foot Ankle Int.

[48] Jerosch J, Schunck J, Abdel-Aziz H (2009). The stop-screw technique. A simple and reliable method in treating flexible flatfoot in children. Foot Ankle Surg.

[49] Nogarin L, Brigantini A, Magnan B, Molinaroli F (1987). Calcaneo-stop: modifiche all’endortesi e alla via chirurgica. Chir Piede.

[50] Frank CB (2004). Ligament structure, physiology and function. J Muscoloskelet Neuronal Interact.

[51] Stagni R, Leardini A, O'Connor JJ, Giannini S (2003). Role of passive structures in the mobility and stability of the human subtalar joint: a literature review. Foot Ankle Int.

[52] De Pellegrin M, Brivio A, Fracassetti D (2010) Calcaneo-stop con vite AO. In: “Il Piede Pediatrico”. Timeo Editore, Bologna, pp 247–256

[53] Rein S, Hagert E, Hanisch U (2013). Immunohistochemical analysis of sensory nerve endings in ankle ligaments: a cadaver study. Cells Tissues Organs.

[54] Rein S, Hanisch U, Zwipp H (2013). Comparative analysis of inter- and intraligamentous distribution of sensory nerve endings in ankle ligaments: a cadaver study. Foot Ankle Int.

[55] Khin MH, Ishii T, Sakane M, Hayashi K (1999). Effect of anaesthesia of the sinus tarsi on peroneal reaction time in patients with functional instability of the ankle. Foot Ankle Int.

